# Association between sleep-disordered breathing and periodontal diseases: A systematic review protocol

**DOI:** 10.3389/fmed.2022.960245

**Published:** 2022-08-08

**Authors:** Danyan Chen, Ziyan Meng, Tingting Zhao, Xueqian Yu, Hong He, Fang Hua, Weili Dong

**Affiliations:** ^1^Hubei-MOST KLOS and KLOBM, School and Hospital of Stomatology, Wuhan University, Wuhan, China; ^2^Department of Periodontology, School and Hospital of Stomatology, Wuhan University, Wuhan, China; ^3^Department of Periodontology, The Affiliated Stomatological Hospital of Nanjing Medical University, Nanjing, China; ^4^Jiangsu Province Key Laboratory of Oral Diseases, Nanjing, China; ^5^Jiangsu Province Engineering Research Center of Stomatological Translational Medicine, Nanjing, China; ^6^Department of Orthodontics, School and Hospital of Stomatology, Wuhan University, Wuhan, China; ^7^Center for Dentofacial Development and Sleep Medicine, School and Hospital of Stomatology, Wuhan University, Wuhan, China; ^8^Library, School and Hospital of Stomatology, Wuhan University, Wuhan, China; ^9^Center for Evidence-Based Stomatology, School and Hospital of Stomatology, Wuhan University, Wuhan, China; ^10^Division of Dentistry, School of Medical Sciences, Faculty of Biology, Medicine and Health, University of Manchester, Manchester, United Kingdom

**Keywords:** sleep-disordered breathing, obstructive sleep apnea, periodontal diseases, systematic review, protocol

## Abstract

**Background:**

Sleep-disordered breathing (SDB) is a chronic sleep-related breathing disorder, considered associated with increased risk of cardiovascular disorders, metabolic disorders, cognitive dysfunction and behavior changes. Periodontal diseases are chronic infectious diseases that are also believed to be associated with cardiovascular diseases, metabolic syndrome and cognitive dysfunction. Several studies have indicated that SDB may be associated with periodontal diseases through certain mechanisms such as inflammation response, oxidative stress and oral dryness. The aim of this systematic review is to explore the association between SDB and periodontal diseases in an integrated approach.

**Materials and Methods:**

This systematic review will include cohort studies, cross-sectional studies and case-control studies that are identified by electronic and manual searches. Electronic searches will be conducted in the following databases: PubMed, Embase, Scopus and Web of Science. Our search will cover articles published from inception of databases to March 2022 without restrictions in language and settings. Pre-determined eligibility criteria include: participants (participants without a history of respiratory diseases, history of periodontal treatment within the past 6 months and history of medication that is known to influence SDB or periodontal diseases); exposure (participants who have been diagnosed with SDB or at high-risk for SDB); comparison (participants without SDB); and outcome (periodontal parameters, such as probing depth, clinical attachment level, bleeding on probing, radiographic bone loss). Two authors will perform study screening and data extraction independently and in duplicate. All discrepancies will be solved by discussion. The methodological quality of included studies will be assessed using the Newcastle-Ottawa Scale.

**Discussion:**

This systematic review will summarize the existing evidence on the association between SDB and periodontal diseases, a topic of controversy and clinical significance. Its findings can provide evidence for the development of relevant prevention and treatment strategies. The results will be disseminated through peer-reviewed journals.

**Systematic review registration:**

https://www.crd.york.ac.uk/PROSPERO/, identifier: CRD42022313024. Registered on March 28th 2022.

## Introduction

Sleep-disordered breathing (SDB) encompasses a spectrum of disorders, including primary snoring, obstructive sleep apnea (OSA) disorders, central sleep apnea (CSA) syndromes, sleep-related hypoventilation and sleep-related hypoxemia (1). Characterized by episodic sleep-related obstruction of the upper airway, OSA is the most common phenotype of SDB (2). Predisposing factors include obesity, tonsillar and adenoid hypertrophy, a short neck and retrognathia (2, 3). As a consequence of sleep apnea, sleep fragmentation results in excessive daytime sleepiness and poor concentration (4). SDB is associated with increased incidence of cardiovascular diseases, neurocognitive deficits and metabolic syndrome (5, 6). The prevalence of SDB ranges widely from 0.7 to 36.5%, depending on gender, age and ethnicity (7–9).

Periodontal diseases mainly include gingivitis and periodontitis, which are a series of chronic inflammatory diseases caused by bacteria in the dental plaque and products of host immune response (10, 11). In 2015–2016 prevalence of periodontitis in Mainland China population aged 35 years or older was almost 90%, and the rate of severe periodontitis was over 30% (12). The periodontal diseases are associated with systemic chronic diseases including diabetes mellitus, atherosclerosis, cardiovascular diseases and metabolic syndrome (13–16). Recently, studies have indicated that periodontal diseases may be associated with SDB (17, 18).

Some potential mechanisms underlying the association between SDB and periodontal diseases have been raised in the literature. Cyclical episodes of hypoxemia-reoxygenation injury in SDB patients can enhance levels of pro-inflammatory cytokines and oxygen free radicals, leading to oxidative stress and inflammation response (2). Likewise, in patients with periodontitis, the inflammatory cytokines have been shown to be elevated (19). The pro-inflammatory cytokines and oxidative stress are responsible for the destruction of periodontal tissue (20). Evidence indicated that inflammation biomarkers related to both conditions include interleukin (IL)-1, IL-6, IL-8, tumor necrosis factor-α (TNF-α), high-sensitivity C-reactive protein (hs-CRP) and others (21). Consequently, the pro-inflammatory immune response may result in a bidirectional link between the two conditions. Another potential mechanism is oral dryness due to mouth breathing. Dry mouth symptoms caused by prolonged oral breathing are common in patients with SDB (22), which leads to greater bacterial colonization and accumulation, as well as increased risk of periodontal diseases (23, 24).

Several studies have reported a significant association between SDB and periodontitis (17, 25, 26). An observational study found that the risk of periodontitis in patients at high risk of OSA was approximately double that at low risk of OSA (27). In addition, a population-based study indicated a lower proportion of OSA in periodontal patients who had undergone either periodontal flap surgery or gingivectomy (28). Conversely, some researchers demonstrated a conflicting conclusion that there was no association between OSA and the prevalence of moderate/severe periodontitis (29). To our knowledge, there is no published systematic review on the relationship between SDB and periodontal diseases. Al-Jewair et al. (30), Lembo et al. (31) and Khodadadi et al. (32) investigated the relationship between OSA and periodontal diseases, but those reviews focused on OSA rather than all sleep-related breathing disorders. Hence, the results of this systematic review will be more comprehensive and detailed.

## Materials and methods

### Protocol and registration

This protocol was written according to the Preferred Reporting Items for Systematic Reviews and Meta-Analyses Protocols (PRISMA-P) checklist. The checklist is displayed in Supplementary file 1. The present systematic review is registered in the PROSPERO database (CRD42022313024).

### Research question

This systematic review summarizes the existing evidence regarding the relationship between SDB and periodontal diseases. The aim is to investigate whether there is an association between various types of SDB and periodontal diseases.

### Criteria for considering studies for this review

#### Types of studies

We will include observational studies using a cohort, case-control, or cross-sectional design that report the correlation between various types of SDB and periodontal diseases. Book chapters, opinion pieces, abstracts, review articles, case report, case series and randomized controlled trials will be excluded.

#### Types of participants

We will include observational studies of participants with and without SDB, diagnosed either objectively with overnight polysomnography (PSG) or subjectively using self-reported questionnaires. SDB is classified into the following categories: OSA, CSA, sleep-related hypoventilation, sleep-related hypoxemia and snoring (1). The apnea-hypopnea index (AHI) is defined as the number of apneas and hypopneas per hour during sleep. The American Academy of Sleep Medicine defines apnea and hypopnea as varying degrees of reduction in respiratory airflow, and these events at least have a span of 10 s (33). OSA is indicated when the PSG results suggest AHI ≥5/h in adult patients or AHI ≥1/h in pediatric patients. CSA is defined as AHI ≥5/h in adult or AHI ≥1/h in children, with ≥50% of these apneas /hypopneas being due to central respiratory events (34, 35). Sleep-related hypoventilation is characterized by an elevated level of PaCO_2_ to a value ≥45 mmHg while asleep or by abnormally increased PaCO_2_ levels compared to those while awake (36). Sleep-related hypoxemia is defined as an arterial oxygen saturation of ≤ 88% for more than 5 min during sleep (1). Snoring is the acoustic phenomena during sleep reported by the affected patient or bed partner. It is an isolated diagnosis without hints for OSA (37). The self-reported questionnaires used to assess risk of SDB include Berlin Questionnaire (BQ), Epworth's Sleepiness Scale (ESS), Pediatric Sleep Questionnaire (PSQ), Stanford Sleepiness Scale, the snoring, tiredness, observed apnea, high BP, BMI, age, neck circumference, and male gender (STOP-Bang) questionnaire, and Apnea Risk Evaluation System (ARES). We will exclude studies in which over 30% participants had a history of respiratory diseases, history of periodontal treatment within the past 6 months or history of medication that is known to influence SDB or periodontal diseases.

#### Types of outcome measures

The selection of periodontal parameters was based on the 2018 Consensus Report of Classification of Periodontal and Peri-implant Diseases and Conditions (38).

*Primary outcome*

- Probing depth (PD)

*Secondary outcomes*

- Clinical attachment level (CAL)
- Bleeding on probing (BOP)
- Radiographic bone loss (RBL)
- Oral hygiene indices (OHI)
- Plaque index (PI)
- Gingival index (GI)
- Gingival recession (GR)
- Inflammation biomarkers (e.g., IL-1, IL-6, TNF-α, hs-CRP).

### Search strategy

We will conduct both electronic searches and manual searches to identify articles related to our research question. Electronic searches without any language and geographic setting restrictions will be conducted in the following four databases: PubMed, Embase, Scopus, and Web of Science (from inception to March 2022). Search strategies combining medical subject headings (MeSH) and free texts will be used according to specifications of each database. Search strategy for PubMed is shown in Table 1 and details for other three databases is depicted in Supplementary file 2. We will also conduct a supplementary manual search by checking the reference lists of key relevant studies and review articles for additional studies that are missed in electronic searches.

**Table 1:** Example search strategy for PubMed.

**Search concept**	
Sleep-disordered breathing	Periodontal diseases
**#1** Sleep Apnea Syndromes [Mesh Terms] **#2** Mouth breathing [Mesh Terms] **#3** (sleep apnea^*^ OR sleep disordered breathing OR sleep-related breathing disorder^*^ OR sleep breathing disorder^*^ OR snoring OR snore OR SDB OR OSA OR OSAS OR OSAHS) [Title/Abstract] **#4** #1 OR #2 OR #3	**#5** Dentistry [Mesh Terms] **#6** Periodontal Diseases [Mesh Terms] **#7** Gingival Diseases [Mesh Terms] **#8** (periodontal disease^*^ OR periodontitis OR gingival disease^*^ OR gingivitis OR periodontal index OR gingival index OR plaque index OR alveolar bone loss OR clinical attachment loss OR periodontal pocket depth^*^ OR tooth mobility OR tooth loss^*^ OR gingival recession* OR dental plaque^*^) [Title/Abstract] **#9** #5 OR #6 OR #7 OR #8

Overall search = #4 AND #9. The

* symbol indicates the variable truncation.

### Data collection and analysis

#### Study selection

Records retrieved from each database will be imported into Endnote X9 and duplicates will be identified and removed. Two authors will independently check the titles and abstracts of retrieved records and exclude articles that clearly do not meet the inclusion criteria. Then we will examine full texts of the remaining articles independently and in duplicate to identify studies that meet the eligibility criteria. Any discrepancy regarding the eligibility of articles will be resolved by discussion among authors. The study selection process will be presented with a flow chart according to the Preferred Reporting Items for Systematic Reviews and Meta-Analyses (PRISMA) checklist (39).

#### Data extraction and management

Two authors will use a piloted data extraction form to collect data from each included study independently and in duplicate. We will resolve discrepancies through discussion and consult a third reviewer if necessary to reach a consensus. If full-texts of included studies lack required information or data, we will contact the corresponding authors and request them to provide. If multiple publications exist for the same study, we will use data from the article that report the latest follow-up or contain the most information. We will record the following data:

- Study characteristics (author; title; publication year; geographical setting; study design; sample size);
- Participant characteristics (number; age; sex; inclusion and exclusion criteria; SDB diagnostic criteria; periodontal parameters);
- Outcome characteristics (primary and secondary outcomes collected; duration of follow-up);
- Other characteristics (funding sources; conflicts of interest).

#### Assessment of risk of bias in included studies

We will use Newcastle-Ottawa Quality Assessment Scale (NOS) (40) to assess the methodological quality of included cohort and case-control studies, and an adapted form of NOS (41) to assess cross-sectional studies. NOS uses a semi quantitative “star system” with a maximum score of 9 to judge studies from three perspectives: selection of study groups, comparability of groups, and ascertainment of outcomes. We will classify the overall risk of bias for each included study as low risk of bias if the NOS score is equal to or >6 and high risk of bias if the score is <6 (42, 43). Two authors will independently assess the included studies and any disagreement will be resolved by discussion with two other experts.

#### Measures of association

For dichotomous outcomes, we will use odd ratios (OR) with 95% confidence intervals (CI) to express estimates of effect. For continuous results measured using the same scale, we will summarize the data with mean difference (MD) and standard deviations (SD). For continuous results measured using different scales or units, we will use standardized mean difference (SMD).

#### Assessment of heterogeneity

Before combining studies in meta-analysis, we will assess clinical heterogeneity by examining the diversity of participants, exposures and outcomes to ensure that the included studies are sufficiently homogeneous to estimate summary effects. We will evaluate statistical heterogeneity with a chi-square test, using *P*-value < 0.1 as indicator of statistically significant heterogeneity. The I square (*I*^2^) statistic will be used to quantify heterogeneity with low values indicating little heterogeneity and high values indicating high heterogeneity:

- 0–40%: low heterogeneity;
- 30–60%: moderate heterogeneity;
- 50–90%: considerable heterogeneity;
- 75–100%: high heterogeneity (44).

#### Assessment of reporting biases

A funnel plot and the Egger's test will be used to assess publication bias of studies if more than 10 studies are included in a meta-analysis (45). If asymmetry is found, we will investigate the possible causes.

#### Data synthesis

When two or more studies provide comparable data, we will perform a meta-analysis to explore the association between SDB and periodontal diseases. Meta-analyses will be performed using the generic inverse variance method with Review Manager 5.3. Where meta-analyses are not possible, we will use a narrative approach to present data on outcome of interest.

#### Subgroup analyses and investigation of heterogeneity

If the included studies can provide adequate data, we will perform subgroup analyses according to the following characteristics:

- Study design (cohort study, case-control study, cross-sectional study);
- Age of participants (children vs. adults);
- Type of SDB (snoring, obstructive sleep apnea disorders, central sleep apnea syndromes, sleep-related hypoventilation, sleep-related hypoxemia).

#### Sensitivity analyses

Sensitivity analyses will be carried out to evaluate the robustness of meta-analysis results. We will repeat the analysis with the adjustment of excluding studies with high risk of bias.

## Discussion

Over the past few years, several studies have investigated the bi-directional relationship between SDB and periodontal diseases, suggesting that the two conditions share some common risk factors (17, 25–27). SDB and periodontal diseases are both associated with some systematic diseases, including cardiovascular diseases, metabolic disorders and cognitive dysfunction (5, 10, 46). Some potential mechanisms have been introduced in the direction of the association between the two diseases. Oxidative stress, inflammation response and oral dryness maybe the bridges between them (20–22, 24). However, other studies have found that SDB was not related to periodontal diseases (29, 47). With increasing complexity of the question, it is necessary to synthesize and update available evidence.

We will conduct this systematic review based on PRISMA guidelines and assess the methodological quality of included studies according to NOS. Strict inclusion and exclusion criteria will be considered, and both electronic and manual searches will be performed to obtain comprehensive data. However, potential limitations of this systematic review are worth noting. Inconsistent sample characteristics and diagnostic methods may result in substantial heterogeneity. If possible, we will perform subgroup analyses to investigate heterogeneity.

In summary, this systematic review will integrate the existing evidence regarding the association between SDB and periodontal diseases, which are important components of population health. Our search strategy has no restriction on age, language or race, which may lead us to find more comprehensive and detailed evidence of the association between SDB and periodontal diseases. Our findings will help to motivate clinicians and policy makers to consider the importance of the association between these two diseases, and will promote prevention and treatment strategies for both diseases. By integrating existing evidence, we may uncover gaps in research of this field, thereby provide guidance for future research.

## Author contributions

DC, ZM, and FH conceived the idea. DC, ZM, TZ, XY, HH, and FH designed methodology. DC, ZM, and XY developed search strategies. DC and ZM drafted the original manuscript. TZ, HH, FH, and WD revised the manuscript. All authors contributed to the article and approved the submitted version.

## Conflict of interest

The authors declare that the research was conducted in the absence of any commercial or financial relationships that could be construed as a potential conflict of interest.

## Publisher's note

All claims expressed in this article are solely those of the authors and do not necessarily represent those of their affiliated organizations, or those of the publisher, the editors and the reviewers. Any product that may be evaluated in this article, or claim that may be made by its manufacturer, is not guaranteed or endorsed by the publisher.
