# Supplementary material for: Association between sleep-disordered breathing and periodontal diseases: A systematic review protocol
Source: Front Med (Lausanne). 2022 Aug 8;9:960245. doi: 10.3389/fmed.2022.960245 (PMC9393785; doi:10.3389/fmed.2022.960245)
Supplement: Supplementary file 2 [file Data_Sheet_1.DOCX]

**Additional file 2**

**Embase search strategy**

**#1** 'sleep disordered breathing'/exp

**#2** 'mouth breathing'/exp

**#3** ('sleep apnea*' OR 'sleep disordered breathing' OR 'sleep-related breathing disorder*' OR 'sleep breathing disorder*' OR 'snoring' OR 'snore' OR 'SDB' OR 'OSA' OR 'OSAS' OR 'OSAHS'):ab,ti

**#4** #1 OR #2 OR #3

**#5** 'dentistry'/exp

**#6** 'periodontal disease'/exp

**#7** 'gingiva disease'/exp

**#8** ('periodontal disease*' OR 'periodontitis' OR 'gingival disease*' OR 'gingivitis' OR 'periodontal index' OR 'gingival index' OR 'plaque index' OR 'alveolar bone loss' OR 'clinical attachment loss' OR 'periodontal pocket depth*' OR 'tooth mobility' OR 'tooth loss*' OR 'gingival recession*' OR 'dental plaque*'):ab,ti

**#9** #5 OR #6 OR #7 OR #8

**#10**  #4 AND #9

**Scopus search strategy**

**#1** TITLE-ABS-KEY ("sleep apnea*" OR "sleep disordered breathing" OR "sleep-related breathing disorder*" OR "sleep breathing disorder*" OR "mouth breathing" OR "snoring" OR "snore" OR "SDB" OR "OSA" OR "OSAS" OR "OSAHS")

**#2** TITLE-ABS-KEY ("dentistry" OR "periodontal disease*" OR "periodontitis" OR "gingival disease*" OR "gingivitis" OR "periodontal index" OR "gingival index" OR "plaque index" OR "alveolar bone loss" OR "clinical attachment loss" OR "periodontal pocket depth*" OR "tooth mobility" OR "tooth loss*" OR "gingival recession*" OR "dental plaque*")

**#3** #1 AND #2

**WOS search strategy**

**#1** TS=((sleep apnea*) OR (sleep disordered breathing) OR (sleep-related breathing disorder*) OR (sleep breathing disorder*) OR (Mouth Breathing) OR (snoring) OR (snore) OR (SDB) OR (OSA) OR (OSAS) OR (OSAHS))

**#2** TS=((dentistry) OR (periodontal diseases) OR (periodontitis) OR (gingival disease*) OR (gingivitis) OR (periodontal index) OR (gingival index) OR (plaque index) OR (alveolar bone loss) OR (clinical attachment loss) OR (periodontal pocket depth*) OR (tooth mobility) OR (tooth loss*) OR (gingival recession*) OR (dental plaque*))

**#3** #1 AND #2
